# Risk Factors for Postoperative Complications After Superomedial Pedicle Reduction Mammaplasty

**DOI:** 10.1093/asjof/ojag141

**Published:** 2026-07-29

**Authors:** Alexandra Boric, Bo Hyun Kong, Ravi Dhawan, Gabriela García Nores, Albert Losken

## Abstract

**Background:**

The superomedial pedicle (SMP) is a widely used reduction mammaplasty technique, but patient-specific risk factors may influence complications.

**Objectives:**

The aim of this study was to quantify postoperative complication rates and identify independent predictors after bilateral SMP breast reduction.

**Methods:**

In this study, the authors retrospectively reviewed 643 consecutive patients undergoing bilateral SMP breast reduction for macromastia from January 2004 to December 2024. Complications were classified as minor (delayed healing, infection, seroma, hematoma, and necrotic events) or major (readmission and/or reoperation). Logistic regression identified predictors, adjusting for age, BMI or obesity class (BMI <30, Classes I-III), smoking, hypertension, and diabetes.

**Results:**

The mean age was 39.3 years (range, 14-74 years), and the mean BMI was 32.5 kg/m^2^ (range, 18.9-58.2 kg/m^2^). The overall complication rate was 22.2% (15.4% minor, 6.8% major). The mean follow-up was 5.5 years. All obesity classes predicted minor complications and delayed healing. Only Classes II to III predicted any complication (all *P* ≤ .008) and infection (all *P* ≤ .025). In continuous BMI models, higher BMI (per 1 kg/m^2^ increase) predicted any complication (*P* = .011), minor complications (*P* = .002), and delayed healing (*P* = .001). Smoking predicted major complications (*P* = .007) and reoperation (*P* = .033), whereas hypertension predicted any complication (*P* = .023) and readmission (*P* = .039). Total resection weight (per 100 g) was not a predictor of most complications, except nipple necrosis (*P* = .001).

**Conclusions:**

The superomedial reduction technique is safe and reproducible even in high-risk patients. Postoperative complications are driven predominantly by patient physiologic risk factors rather than resection volume.

**Level of Evidence: 3 (Therapeutic):**

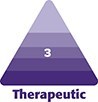

Breast reduction is a commonly performed procedure that reliably improves symptoms and patient satisfaction, with 76,734 cases performed in the United States in 2024.^1-13^ Macromastia is associated with chronic pain, physical limitations, intertriginous rashes, and impaired quality of life.^6,8,9,11-14^ Multiple pedicle designs have been described, each with distinct advantages in terms of nipple–areolar complex (NAC) viability, breast shape, and technical reproducibility. Among these, the superomedial pedicle (SMP) is widely favored because of its robust vascular supply, favorable upper pole projection, and reliable aesthetic outcomes.^14-17^

Despite widespread adoption of the SMP, postoperative complications remain a concern. Reported complication rates in the reduction mammaplasty literature vary widely, in part because of heterogeneity in technique, patient selection, and outcome definitions. Previous studies often aggregate multiple pedicle types, making it difficult to distinguish technique-related determinants from patient-related determinants of risk during counseling and perioperative optimization. Accordingly, it remains important to clarify whether complications following SMP reduction are primarily driven by resection volume and technical factors or by patient risk profiles.

The primary objective of this study was to characterize postoperative complication rates in a large cohort of patients undergoing bilateral SMP breast reduction. The secondary objective was to identify independent predictors of postoperative complications using multivariable logistic regression, with particular focus on BMI, smoking, hypertension, diabetes, and total resection weight.

## METHODS

### Study Population

**Figure 1.**
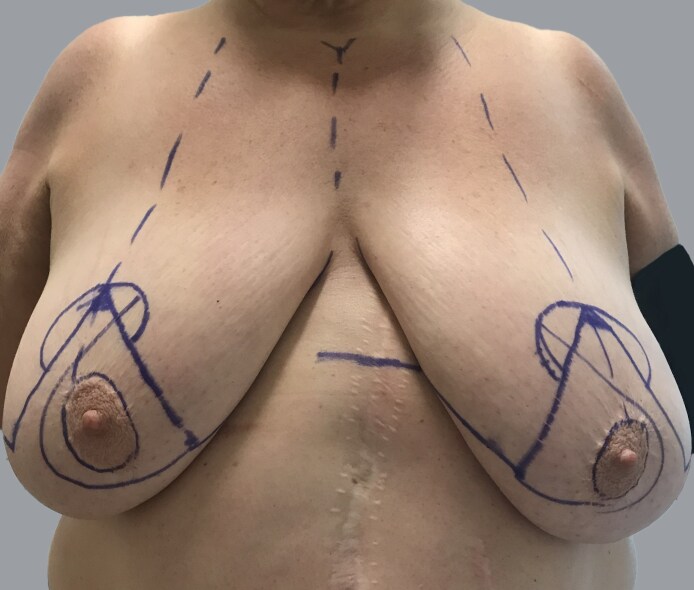
Preoperative markings for superomedial pedicle breast reduction in a 64-year-old woman with a BMI of 42.7.

**Figure 2.**
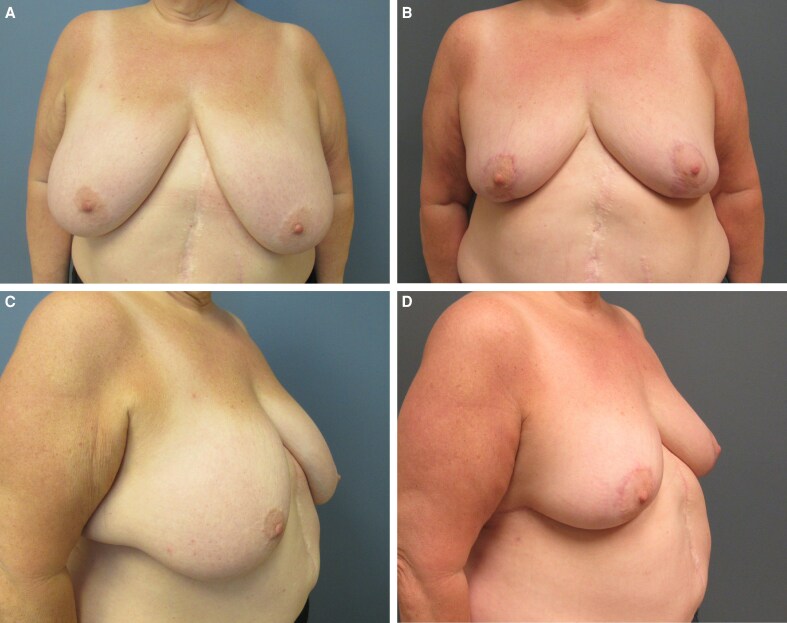
Preoperative and postoperative photographs of a 64-year-old woman who underwent superomedial breast reduction. Anterior view at (A) the preoperative time point and (B) the 12-month postoperative time point. Anterolateral view at (C) the preoperative time point and (D) the 12-month postoperative time point.

This retrospective cohort study included all patients who underwent bilateral SMP breast reduction for symptomatic macromastia by a single surgeon between January 2004 and December 2024 at the Emory hospitals. This study was approved by the Emory University IRB. The electronic medical record and operative reports were reviewed to identify eligible patients; those who underwent unilateral procedures, non-SMP techniques, or concomitant oncologic resections were excluded. A total of 643 consecutive patients met inclusion criteria and were included in the final analysis. Representative preoperative markings (Figure 1) and corresponding preoperative (without markings) and 12-month postoperative clinical photographs (Figure 2) from a patient within the study cohort illustrate typical operative planning and postoperative breast shape.

### Data Collection

Collected variables were entered into a secure internal database. Abstracted data included patient demographics (age and preoperative BMI), comorbidities (smoking status and history of diabetes or hypertension), operative characteristics (pedicle selection and right and left breast resection weights), and the most recent in-person follow-up date. Postoperative events were identified through review of outpatient clinic notes, emergency department encounters, and hospital records, with all complications, readmissions, and reoperations recorded along with their indication and timing. Patients who reported smoking were counseled preoperatively regarding the increased risk of wound-healing complications and were encouraged to pursue smoking cessation before surgery. Smoking was not considered an absolute contraindication.

Events were classified as minor or major based on clinical impact and need for invasive intervention. Minor complications included delayed wound healing (persistent wound breakdown or drainage requiring dressing changes documented at ≥2 consecutive clinic visits without need for closure or debridement), infection treated with oral antibiotics alone, seroma or hematoma managed nonoperatively, total or partial NAC necrosis requiring dressing changes, fat necrosis requiring in-office debridement, and skin necrosis.

Major complications were defined as readmission or reoperation for a surgery-related complication within 90 days postoperatively. Indications for reoperation included excisional debridement for skin necrosis, excisional debridement for delayed wound healing, hematoma/seroma drainage, nipple discomfort or NAC necrosis debridement, fat necrosis excision, functional complication, infection requiring operative washout, and an “other” category (eg, breast trauma and seroma).

Procedures performed after 90 days or for aesthetic or revisional reasons were classified as secondary surgeries. Indications for secondary surgery included asymmetry, ptosis, scarring, residual breast tissue, and cosmetic dissatisfaction.

### Statistical Analysis

Descriptive statistics were used to summarize patient demographics, comorbidities, and operative characteristics. Continuous variables are reported as means with standard deviations (SDs) and ranges, and categorical variables as frequencies with percentages. Unadjusted comparisons of complication rates across BMI classes were performed using Pearson χ^2^ test.

Multivariable logistic regression was used to identify independent predictors of each outcome. Covariates included age (years), current smoking status (current or use within 2 months of surgery), hypertension, and diabetes. BMI was evaluated in separate models as (1) a continuous variable (per 1 kg/m^2^), (2) a binary variable (obesity defined as BMI ≥30 kg/m^2^), and (3) obesity classes (Class I 30.0-34.9, Class II 35.0-39.9, Class III ≥40.0; reference BMI <30) to avoid collinearity between BMI and obesity. A complete-case analysis was used for multivariable models, excluding patients with missing data in any covariate or outcome variable.

Adjusted odds ratios (aORs) with 95% CIs and 2-sided *P*-values were reported. *P*-values for regression coefficients were derived from Wald χ^2^ test with robust (Huber–White/sandwich) standard errors. For BMI class models, a test for trend (*P*-trend) was performed by modeling BMI class as an ordinal predictor (<30, Class I, Class II, and Class III) in the adjusted model. Statistical significance was defined as *P* < .05. Analyses were performed using Microsoft Excel (Microsoft Corporation, Redmond, WA), IBM SPSS Statistics Version 27 (IBM Corporation, Armonk, NY), and Julius AI (Julius AI Inc., San Francisco, CA).

## RESULTS

### Patient Characteristics

A total of 643 patients undergoing bilateral SMP breast reduction met inclusion criteria. The mean age at surgery was 39.3 years (SD 14.2; range, 14-74 years). BMI was available for 626 of 643 patients. The mean BMI was 32.5 kg/m^2^ (SD 6.2; range, 18.9-58.2 kg/m^2^), and 63.4% (*n* = 397) of patients met criteria for obesity. BMI classes were distributed as follows: BMI <30 (*n* = 229, 36.6%); Class I: 30.0 to 34.9 kg/m^2^ (*n* = 189, 30.2%); Class II: 35.0 to 39.9 kg/m^2^ (*n* = 136, 21.7%); and Class III: ≥40.0 kg/m^2^ (*n* = 72, 11.5%). Diabetes was present in 51 patients (7.9%), hypertension in 143 patients (22.2%), and current smoking in 62 patients (9.6%).

The mean right breast specimen weight was 819.6 g (SD 388.1; range, 59-3396 g), and the mean left breast specimen weight was 831.6 g (SD 383.3; range 77-3016 g). The mean total specimen weight was 1650.1 g (SD 750.8; range, 136-6412 g). The mean right nipple-to-notch distance was 33.2 cm (SD 4.8; range, 12.0-63.5 cm; *n* = 585) and left was 33.4 cm (SD 4.8; range, 12.0-59.0 cm; *n* = 584). The skin excision pattern used was primarily Wise pattern (*n* = 630, 97.5%). The mean duration of chart-based postoperative surveillance, measured from the surgery date to the last in-person follow-up appointment, was 5.5 years (SD 4.3, median 4.1, range 0.9-21.0). Patient demographics and operative characteristics are summarized in Table 1.

**Table 1.** Patient Demographics and Operative Characteristics in the Superomedial Breast Reduction Cohort (*N* = 643)

Variable	Value	Range
Age, years (mean ± SD)	39.3 ± 14.2	14-74
BMI, kg/m^2^ (mean ± SD)	32.5 ± 6.2	18.9-58.2
Obesity (BMI ≥30), *n* (%)	397 (63.4)	
Obesity class, *n* (%)		
Nonobese (BMI <30)	229 (36.6)	
Class I	189 (30.2)	
Class II	136 (21.7)	
Class III	72 (11.5)	
Smokers, *n* (%)	62 (9.6)	
Hypertension prevalence, *n* (%)	143 (22.2)	
Diabetes mellitus prevalence, *n* (%)	51 (7.9)	
Total resection weight, g (mean ± SD)		
Total	1650.1 ± 750.8	136-6412
Left	831.6 ± 383.3	77-3016
Right	819.6 ± 388.1	59-3396
Nipple-to-notch, cm (mean ± SD)		
Left	33.4 ± 4.8	12.0-59.0
Right	33.2 ± 4.8	12.0-63.5
Follow-up duration, years (mean ± SD)	5.5 ± 4.3	0.9-21.0

BMI data were available for 627 patients. Nipple-to-notch measurements were available for 585 right breasts and 584 left breasts. Obesity classes were defined as follows: Class I (BMI 30.0-34.9 kg/m^2^), Class II (BMI 35.0-39.9 kg/m^2^), and Class III (BMI ≥40.0 kg/m^2^). SD, standard deviation.

### Postoperative Outcomes

The overall complication rate was 22.2% (143/643), including a minor complication rate of 15.4% (99/643) and a major complication rate of 6.8% (44/643). Among minor complications, delayed wound healing was most common (*n* = 46, 7.2%), followed by infection (*n* = 37, 5.8%), seroma (*n* = 18, 2.8%), hematoma (*n* = 9, 1.4%), NAC necrosis (*n* = 6, 0.9%), and fat necrosis (*n* = 8, 1.2%).

Major complications were largely attributable to reoperations (31/643, 4.8%). Among the 31 patients who underwent reoperation, a total of 34 indications were recorded, most commonly hematoma drainage (14/34, 41.2%), followed by excisional debridement for delayed wound healing (9/34, 26.5%). Other indications included “other” (4/34, 11.8%; 3 for seroma drainage and 1 for wound dehiscence following breast trauma), fat necrosis (3/34, 8.8%), debridement of skin necrosis (2/34, 5.9%), and infection requiring operating-room washout (2/34, 5.9%). Readmissions occurred in 21 patients (21/643, 3.3%). There were 31 total readmission indications, most commonly infection requiring oral or intravenous antibiotics (11/31, 35.5%), followed by hematoma (5/31, 16.1%) and seroma (5/31, 16.1%). Other indications included “other” (4/31, 12.9%; 3 cases of observation and 1 case of tachycardia) and wound dehiscence (3/31, 9.7%). Less common indications were severe pain (1/31, 3.2%), delayed wound healing (1/31, 3.2%), and thrombosis/pulmonary embolism (1/31, 3.2%).

Secondary surgeries were performed in 27 patients. Among these patients, 37 total indications were recorded. The most common indication was scarring (19/37, 51.4%), followed by fat necrosis (7/37, 18.9%). Less common indications included cosmetic dissatisfaction (3/37, 8.1%), asymmetry (2/37, 5.4%), regained breast volume (5.4%, 2/37), and excisional debridement for delayed wound healing (2/37, 5.4%). Rare indications included residual breast mass (1/37, 2.7%) and debridement of necrotic skin/subcutaneous tissue (1/37, 2.7%). Postoperative outcomes are summarized in Table 2.

**Table 2.** Postoperative Complications and Reinterventions Following Superomedial Pedicle Breast Reduction

Variable	% (*n*/*N*)
Overall complications	22.2 (143/643)
Minor	15.4 (99/643)
Delayed wound healing	7.2 (46)
Infection	5.8 (37)
Seroma	2.8 (18)
Hematoma	1.4 (9)
Fat necrosis	1.2 (8)
Nipple necrosis	0.9 (6)
Major	6.8 (44/643)
Reoperations	4.8 (31/643)
Hematoma drainage	41.2 (14/34)
Excisional debridement, DWH	26.5 (9/34)
Other	11.8 (4/34)
Fat necrosis	8.8 (3/34)
Excisional debridement, SFN	5.9 (2/34)
Infection	5.9 (2/34)
Readmissions	3.3 (21/643)
Infection	35.5 (11/31)
Hematoma	16.1 (5/31)
Seroma	16.1 (5/31)
Other	12.9 (4/31)
Severe pain	3.2 (1/31)
Delayed wound healing	3.2 (1/31)
Thrombosis/PE	3.2 (1/31)
Secondary Surgeries	4.2 (27/643)
Scarring	51.4 (19/37)
Fat necrosis	18.9 (7/37)
Cosmetic dissatisfaction	8.1 (3/37)
Asymmetry	5.4 (2/37)
Excisional debridement, DWH	5.4 (2/37)
Regained breast volume	5.4 (2/37)
Excisional debridement, SFN	2.7 (1/37)
Residual breast tissue	2.7 (1/37)

Minor complications did not require readmission or reoperation, whereas major complications were defined as readmission and/or reoperation within 90 days. Complication subtypes were not mutually exclusive; patients could experience more than one complication. Indications for reoperation, readmission, and secondary surgery are reported as the number of indications per total indications rather than per patient, because multiple indications could be recorded for a single event. Therefore, the number of indications may exceed the number of readmission or reoperation events. DWH, delayed wound healing; PE, pulmonary embolism; SFN, skin flap necrosis.

### Multivariable Analysis of Risk Factors

On multivariable logistic regression adjusting for age, smoking, hypertension, and diabetes, both obesity and BMI were significant predictors of postoperative morbidity. Obesity was independently associated with higher odds of any complication (aOR 1.89, 95% CI, 1.21-2.93, *P* = .005) and any minor complication (aOR 2.86, 95% CI, 1.66-4.95, *P* < .001), and it was a particularly strong predictor of wound-healing morbidity, including delayed wound healing (aOR 4.81, 95% CI, 1.85-12.50, *P* = .001) and postoperative infection requiring oral antibiotics (aOR 2.41, 95% CI, 1.03-5.64, *P* = .043). In parallel, higher BMI (per 1 kg/m^2^ increase) was independently associated with any complication (aOR 1.05, 95% CI, 1.01-1.08, *P* = .011), any minor complication (aOR 1.06, 95% CI, 1.02-1.10, *P* = .002), and delayed wound healing (aOR 1.08, 95% CI, 1.03-1.13, *P* = .001). Hypertension was also independently associated with any complication (aOR 1.77, 95% CI, 1.08-2.90, *P* = .023) and was a predictor of readmission (aOR 3.22, 95% CI, 1.06-9.76, *P* = .039). The most common indication for readmission among hypertensive patients was infection, accounting for 7 of 20 readmission indications (35.0%). Smoking independently predicted any major complication (aOR 3.05, 95% CI, 1.36-6.86, *P* = .007) and reoperation (aOR 2.78, 95% CI, 1.09-7.11, *P* = .033). The most common indication for reoperation in smokers was wound-healing debridement (4/8, 50.0%; 3 because of delayed wound healing and 1 because of skin necrosis), followed by fat necrosis (2/8, 25.0%).

Total resection weight (per 100 g) was evaluated as a predictor of postoperative complications. On adjusted analysis, resection weight was not associated with overall complications (aOR 1.01, 95% CI, 0.98-1.04; *P* = .583) or with any minor complication (aOR 1.02, 95% CI, 0.98-1.06; *P* = .306). Similarly, resection weight was not associated with seroma (aOR 0.99, 95% CI, 0.92-1.07; *P* = .808), hematoma (aOR 0.93, 95% CI, 0.84-1.03; *P* = .167), skin necrosis (aOR 1.04, 95% CI, 0.95-1.14; *P* = .389), delayed wound healing (aOR 1.04, 95% CI, 1.00-1.09; *P* = .066), infection (aOR 1.02, 95% CI, 0.97-1.07; *P* = .450), and symptomatic fat necrosis (aOR 1.02, 95% CI, 0.93-1.12; *P* = .708). The exception was NAC necrosis, for which higher resection weight was associated with increased odds (aOR 1.21, 95% CI, 1.07-1.36; *P* = .002). Resection weight was also not associated with major complications (aOR 1.02, 95% CI, 0.97-1.07; *P* = .449), including readmission (aOR 0.97, 95% CI, 0.91-1.04; *P* = .396) or reoperation (aOR 1.02, 95% CI, 0.96-1.08; *P* = .541). Independent predictors of postoperative complications are summarized in Table 3.

**Table 3.** Multivariable Predictors of Postoperative Complications Following Superomedial Pedicle Breast Reduction

Predictor	Outcome	aOR	95% CI	*P*-value
**Obesity (BMI** ≥**30)**	Any complication	1.89	1.21-2.93	.005*
	Any minor complication	2.86	1.66-4.95	<.001*
	Delayed wound healing	4.81	1.85-12.50	.001*
	Infection	2.41	1.03-5.64	.043*
**BMI (per 1 kg/m^2^)**	Any complication	1.05	1.01-1.08	.011*
	Any minor complication	1.06	1.02-1.10	.002*
	Delayed wound healing	1.08	1.03-1.13	.001*
**Hypertension**	Any complication	1.77	1.08-2.90	.023*
	Readmission	3.22	1.06-9.76	.039*
**Smoking**	Any major complication	3.05	1.36-6.86	.007*
	Reoperation	2.78	1.09-7.11	.033*
**Total resection weight (per 100 g)**	Nipple necrosis	1.21	1.07-1.36	.002*

Adjusted odds ratios (aORs) with 95% CIs are reported. Obesity was defined as BMI ≥ 30 kg/m^2^. Obesity and BMI were evaluated in separate models to avoid collinearity. All models were adjusted for age, smoking, hypertension, and diabetes, except for the predictor of interest. Continuous predictors are reported per unit increase (BMI per 1 kg/m^2^; total resection weight per 100 g). Patients with missing data for any covariate or outcome were excluded from multivariable analyses. Statistically significant values (*P* < .05) are indicated by an asterisk.

### BMI Stratification by Obesity Class

In an exploratory stratified analysis among patients with available BMI data (*n* = 626), complication rates increased with higher obesity class. The rate of any complication rose from 14.4% (33/229) in patients with BMI <30 to 19.7% (37/188), 28.7% (39/136), and 30.1% (22/73) in Classes I, II, and III, respectively (*P* = .002). Minor complications followed a similar pattern, occurring in 7.9%, 15.4%, 24.3%, and 24.7% across increasing obesity classes (*P* < .001). Delayed wound healing also increased progressively (2.2%, 7.4%, 11.8%, and 13.7%; *P* < .001), as did infection treated with oral antibiotics alone (3.1%, 4.8%, 9.6%, and 11.0%; *P* = .016). In contrast, major complications did not differ across BMI classes (*P* = .81).

In multivariable models adjusted for age, smoking, hypertension, and diabetes (reference BMI <30), obesity Classes II and III were associated with increased odds of any complication (Class II: aOR 2.32 [1.36-3.94], *P* = .002; Class III: aOR 2.37 [1.25-4.48], *P* = .008), whereas Class I was not (aOR 1.46 [0.87-2.45], *P* = .154). For minor complications, all obesity classes were associated with increased odds (Class I: aOR 2.12 [1.13-3.97], *P* = .019; Class II: aOR 3.66 [1.95-6.87], *P* < .001; Class III: aOR 3.64 [1.74-7.60], *P* < .001). Among specific minor outcomes, delayed wound healing was significantly increased across all obesity classes (Class I: aOR 3.53 [1.24-10.04], *P* = .018; Class II: aOR 5.78 [2.04-16.31], *P* < .001; Class III: aOR 6.75 [2.18-20.95], *P* < .001). In contrast, infection treated with oral antibiotics alone was increased in Classes II and III but not Class I (Class I: aOR 1.56 [0.57-4.30], *P* = .389; Class II: aOR 3.12 [1.20-8.14], *P* = .020; Class III: aOR 3.43 [1.17-10.11], *P* = .025). Adjusted associations between obesity class and postoperative outcomes are summarized in Table 4.

**Table 4.** Multivariable Predictors of Postoperative Complications by Obesity Class Following Superomedial Pedicle Breast Reduction

Outcome	aOR	95% CI	*P-*value	*P*-trend
Any complication (minor or major)				<.001*
Class I	1.46	0.87-2.45	.154	
Class II	2.32	1.36-3.94	.002*	
Class III	2.37	1.25-4.48	.008*	
Any minor complication				<.001*
Class I	2.12	1.13-3.97	.019*	
Class II	3.66	1.95-6.87	<.001*	
Class III	3.64	1.74-7.60	<.001*	
Delayed wound healing				<.001*
Class I	3.53	1.24-10.04	.018*	
Class II	5.78	2.04-16.31	<.001*	
Class III	6.75	2.18-20.95	<.001*	
Infection				.007*
Class I	1.56	0.57-4.30	.389	
Class II	3.12	1.20-8.14	.020*	
Class III	3.43	1.17-10.11	.025*	
Any major complication				.716
Class I	0.80	0.35-1.82	.590	
Class II	1.10	0.47-2.53	.831	
Class III	1.16	0.42-3.19	.767	

Adjusted odds ratios (aORs) with 95% CIs are reported from multivariable logistic regression models. The reference category was BMI < 30 kg/m^2^. Obesity classes were defined as Class I (30.0-34.9 kg/m^2^), Class II (35.0-39.9 kg/m^2^), and Class III (≥40.0 kg/m^2^). Models were adjusted for age, smoking, hypertension, and diabetes. *P*-trend represents BMI class modeled as an ordinal variable. Statistically significant values (*P* < .05) are indicated by an asterisk.

## DISCUSSION

The SMP breast reduction remains a reliable technique with manageable postoperative complications. The central finding of this study was that patient physiologic risk profiles, rather than resection volume, were the primary drivers of postoperative complications. Obesity was strongly associated with delayed wound healing and postoperative infection, smoking significantly increased the likelihood of reoperation, and hypertension emerged as the strongest predictor of readmission. In contrast, total specimen weight was not independently associated with minor, major, or overall complications.

Although obesity is often associated with increased comorbidities and should be considered within the context of a holistic evaluation, it remains a risk factor in many breast reduction studies.^17-19^ Previous work in breast reconstruction populations has demonstrated significantly higher complication rates among obese patients compared with nonobese patients, even after adjustment for comorbidities, underscoring the independent contribution of obesity to wound-healing morbidity and infection risk.^17^ Within SMP-specific reduction mammaplasty series, increased BMI has similarly been associated with higher complication rates.^18,19^ However, comparative analyses across multiple pedicle techniques suggest the SMP is associated with lower overall complication rates and may perform reliably across BMI strata.^20-24^ Importantly, these comparative findings reflect technique-level differences and do not preclude residual patient-level risk within a given technique.

Obesity and BMI were both significant predictors of postoperative morbidity in our cohort. In separate multivariable models, both obesity and increasing BMI were independently associated with higher odds of any complication, any minor complication, and delayed wound healing, whereas only obesity was independently associated with postoperative infection requiring oral antibiotics. These findings are supported by previous literature demonstrating that patients with elevated BMI (including those meeting criteria for obesity) who undergo reduction mammaplasty are more likely to experience delayed healing, wound dehiscence, and infection.^25-32^ A recent systematic review and meta-analysis encompassing over 71,000 reduction mammaplasty patients found that obesity (BMI ≥ 30 kg/m^2^) was significantly associated with higher risks of delayed wound healing (OR = 1.93, 95% CI, 1.03-3.63, *P* = .041; *I*^2^ = 0.0%), wound infection (OR = 1.54, 95% CI, 1.29-1.84, *P* = .000; *I*^2^ = 0.0%), and dehiscence (OR = 2.19, 95% CI, 1.27-3.78, *P* = .005; *I*^2^ = 60.8%).^26^ In a retrospective analysis of 453 consecutive reduction mammaplasty cases, minor complications occurred in 40.5% of patients, and those with minor complications had a higher mean BMI than those without complications (30.2 vs 28.0; *P* < .001); on multivariable analysis, BMI was the only significant predictor of minor complications (*P* < .001), which is mirrored in our study.^30^ This relationship is biologically plausible and supported by established evidence demonstrating impaired tissue perfusion, altered inflammatory response, collagen dysregulation, and increased mechanical stress on wounds in obese patients.^33,34^ In the context of reduction mammaplasty, increased skin tension across the closure and larger surface areas at risk may further accentuate the effect of elevated BMI on wound-healing dynamics and increase risk of infection. From a clinical perspective, obese patients should receive targeted preoperative counseling about their elevated risk of wound-related issues and possible prolonged dressing care. Surgeons may consider technique modifications to implement tension-reducing strategies, and postoperative protocols emphasizing close wound surveillance and early intervention for minor breakdowns. Although strict BMI cutoffs remain controversial, these data provide a quantitative framework for shared decision making.

In BMI-stratified analyses, we observed a clear dose–response relationship between increasing obesity class and postoperative morbidity. Unadjusted rates of any complication increased from 14.4% in patients with BMI <30 to 19.7%, 28.7%, and 30.1% in obesity Classes I to III, respectively, driven primarily by minor complications (7.9% vs 15.4%, 24.3%, and 24.7%). In adjusted models, obesity Classes II and III—but not Class I—were associated with increased odds of any complication, whereas all obesity classes were associated with increased odds of minor complications and delayed wound healing; infection risk was significantly increased only for Classes II and III. Notably, major complications did not differ by obesity class, suggesting that increasing BMI primarily increases wound-healing morbidity rather than reoperation/readmission risk. These findings are contrasted with a superomedial short-scar series of 236 patients that reported increasing complication rates across comparable BMI strata without reaching statistical significance (*P*-trend = .145), likely reflecting limited power; our larger cohort confirms and better characterizes this gradient of risk within SMP reduction mammaplasty.^35^

Tobacco use is well documented to increase postoperative morbidity and major complications in plastic surgery, including an elevated risk of reoperation.^36-38^ In our cohort, smoking was an independent predictor of reoperation, consistent with previous reduction mammaplasty-specific studies demonstrating a significant association between tobacco use and increased return-to-operating-room rates.^39,40^ This may be explained by nicotine and carbon monoxide's effects on impairing microvascular perfusion and collagen deposition, including increased fat necrosis and delayed healing rates in smokers and occasionally necessitating operative intervention.^40-43^ Large national database analyses have similarly identified active smoking as an independent predictor of overall (OR 1.7; *P* < .001) and major surgical complications (defined as deep infection and return to the operating room; OR 2.7; *P* < .001) following reduction mammaplasty.^39^ Procedure-specific analyses of aesthetic surgery outcomes likewise demonstrate increased odds of major postoperative complications, including reoperation within 30 days after breast reduction (OR 1.58; 95% CI, 1.08-2.32; *P* = .02).^40^ Although smoking has also been associated with increased infection risk in multiple studies, we did not observe an independent association between smoking and postoperative infection in our adjusted models.^29,39,44-46^ These data reinforce best practices emphasizing strong encouragement of smoking cessation as early as possible before elective reduction mammaplasty. Although smoking cessation is always recommended, the SMP technique might be more reliable than other techniques in this setting given the shorter pedicle and limited skin undermining. Counseling patients that smoking not only increases complication risk but also doubles their chance of returning to the operating room may be a powerful motivator for behavior change.

Hypertension has been inconsistently associated with hospital readmission in previous breast surgery studies.^47-49^ In a multi-institutional American College of Surgeons National Surgical Quality Improvement Program study of 54,336 patients undergoing elective aesthetic breast procedures, breast reduction accounted for 83.5%. They found that medically treated hypertension was independently associated with increased odds of the composite outcome encompassing reoperation, readmission, or unplanned readmission (OR 1.5, 95% CI, 1.3-1.8, *P* < .001) as well as overall complications (OR 1.2, 95% CI, 1.0-1.3, *P* = .0079) following breast reduction.^48^ Similarly, in a large systematic review and meta-analysis evaluating predictors of complications following breast reconstruction following mastectomy, hypertension was associated with increased risk of 0- to 90-day readmission (OR 1.65, 95% CI, 1.06-2.57, *P* < .03), alongside obesity, diabetes, and smoking.^47^ Consistent with these broader analyses, in our cohort, hypertension emerged as the strongest independent predictor for any complication and readmission, with infection representing the most frequent indication for return to care. This may reflect a broader association between chronic vascular disease, immune dysregulation, and impaired soft-tissue perfusion that predisposes hypertensive patients to infectious and wound-related complications. Taken together, these data suggest that hypertension is not simply a background comorbidity but a meaningful modifier of postoperative risk in breast-reduction patients. Preoperative optimization of blood pressure and close early postoperative monitoring of hypertensive patients, particularly with a low threshold to evaluate for infection, may therefore help reduce readmissions and downstream morbidity in this population. of cases

In previous studies, the weight of resected breast tissue has been correlated with an increased complication rate.^18,19,39,50-52^ It is often used as a proxy for breast size with the thought being that larger breasts have a higher risk of complications. A 2006 study identified resection weight as the sole independent predictor of increased complication risk, noting that with each 10-fold increase in resection weight, the risk of complications increased 4.8-fold, and the risk of delayed healing increased 11.6-fold.^50^ Likewise, Palve et al demonstrated that resected tissue weight ≥650 g per breast was independently associated with higher complication rates.^51^ Previous SMP-specific series have also linked resection weight to postoperative morbidity. Morrison et al reported that, with each additional gram of reduction weight, the odds of a surgical complication increased by 1.001, and Darras et al described statistically significant but weak correlations between excised tissue mass and complication rates in their meta-analysis of SMP reductions.^18,19^ However, other SMP-focused data suggest that larger resections do not necessarily translate to higher rates of NAC necrosis. In a retrospective series of SMP reductions (135 breasts/70 patients), Brownlee et al reported an overall NAC necrosis rate of 0.7% per breast, with 0% necrosis when ≤1200 g was resected and 2.3% when >1200 g was resected, a difference that was not statistically significant (*P* = .32).^53^ In our cohort, resection weight was independently associated with NAC necrosis; however, this finding should be interpreted cautiously given the small number of necrosis events (*n* = 6). Notably, specimen weight was not associated with other complication outcomes in this dataset. Although some patients are not ideal candidates for the SMP technique given longer nipple-to-notch distances, it does remain a reliable technique even in patients who require larger resection volumes.^21,54-56^ The data suggest that the SMP can safely support a wide range of resection volumes when applied thoughtfully. Risk stratification and perioperative counseling should therefore focus more heavily on patient-level factors, such as obesity, smoking, hypertension, and diabetes rather than on resection weight alone.

### Limitations

This study represents a large single-surgeon cohort of 643 patients undergoing SMP breast reduction, providing technical consistency and reducing variability related to operative technique. Longitudinal follow-up averaging more than 5 years allowed capture of delayed complications and secondary surgical interventions.

Several limitations warrant consideration. The single-surgeon, high-volume design, while enhancing internal consistency, inherently limits generalizability; outcomes may differ in multisurgeon or lower-volume practice settings with greater heterogeneity in technique, perioperative management, and postoperative surveillance. The retrospective design introduces the possibility of unmeasured confounding, including factors such as nutritional status, smoking intensity, and adherence to postoperative instructions.

Complication ascertainment relied on documentation within the institutional medical record and may therefore underestimate minor events managed outside the health system, which may partially explain the relatively modest minor complication rate observed compared with some previous reduction mammaplasty series. Although mean follow-up exceeded 5 years, the timing of minor complications relative to the index operation was not uniformly available for stratified analysis; however, most surgical morbidity following reduction mammaplasty is expected to occur in the early postoperative period. Smoking was defined as current or recent use within 2 months of surgery; data on smoking intensity, cumulative exposure, and duration of use were not consistently available, and residual confounding related to dose-dependent effects is possible. Similarly, although hypertension emerged as a strong independent predictor of readmission, data distinguishing well controlled from poorly controlled hypertension were unavailable, and differences in blood pressure control may further modify postoperative risk. Accordingly, extended follow-up in this study primarily facilitated identification of delayed presentations and secondary procedures rather than incident early complications.

### Future Directions

Future studies should prioritize prospective, multicenter investigations to validate these findings across diverse practice settings. Standardized definitions and reporting of complications, comorbidities, and operative variables will enable meaningful comparisons while incorporating more detailed patient-level data (eg, smoking intensity and cessation interval, nutritional status, glycemic control, and adherence factors) often incompletely captured in retrospective review. Further work is needed to define clinically relevant risk thresholds and quantify the effects of preoperative optimization on postoperative outcomes.

In parallel, future research should also strengthen outcome ascertainment to minimize under-capture of minor events managed outside the health system and expand endpoints beyond complications to include patient-reported outcomes, aesthetic assessments, and revision surgery patterns. Although the SMP is traditionally associated with improved superior pole projection, aesthetic outcomes were not formally evaluated in this study, and maintenance of superior pole fullness in large-volume reductions may be influenced by tissue weight and long-term settling. Future studies using objective aesthetic measures may help clarify whether these theoretical advantages persist across varying breast sizes and resection volumes.

Finally, comparative and interventional studies evaluating targeted perioperative strategies—such as smoking cessation, blood pressure optimization, and enhanced wound surveillance—may help reduce morbidity and clarify technique-specific advantages. Additional head-to-head comparisons of superomedial and alternative pedicle designs in higher-risk populations may further define technique-specific outcomes.

## CONCLUSIONS

In this large series of patients undergoing bilateral SMP breast reduction, postoperative complication patterns were driven predominantly by patient physiologic risk profiles rather than resection volume. Obesity significantly increased the risk of delayed wound healing and superficial infection. Smoking approximately doubled the likelihood of reoperation, often for fat necrosis and perfusion-related complications. Hypertension was the strongest predictor of readmission, most commonly for postoperative infection. Total specimen weight was not associated with minor, major, or overall complication rates; however, increasing resection volume independently predicted NAC necrosis, suggesting a volume-related perfusion vulnerability of the NAC in select patients. These findings emphasize the importance of targeted preoperative counseling, optimization of modifiable risk factors including obesity, smoking, and hypertension, and appropriate expectation setting in patients undergoing reduction mammaplasty. By focusing on modifiable patient factors rather than resection volume, surgeons can better individualize risk, improve perioperative management, and enhance the safety of SMP breast reduction.
